# Therapeutic Effects of Spent Ground Coffee Oil on Nociception, Anxiety- and Depression-Like Behaviors, and Inflammatory Mechanisms in a CFA-Induced Intraplantar Inflammation Model in Mice

**DOI:** 10.1007/s12035-026-06069-3

**Published:** 2026-07-23

**Authors:** Elisa Mitkus Flores Lins, Anelise Leal Vieira Cubas, Bruna Hoffmann de Oliveira, Eduarda Behenck Medeiros, Khiany Mathias, Scheila Iria Kraus, Daniel Fernandes Martins, Josiel Mileno Mack, Anna Paula Piovezan, Fabricia Petronilho, Josiane Budni, Franciane Bobinski

**Affiliations:** 1 Experimental Neuroscience Laboratory (LaNEx), Postgraduate Program in Health Sciences, University of South Santa Catarina (UNISUL), Avenida Pedra Branca 25, Cidade Universitária, Palhoça, SC 88137-270 Brazil; 2 Postgraduate Program in Administration, University of South Santa Catarina (UNISUL), Avenida Pedra Branca 25, Cidade Universitária, Palhoça, SC 88137-270 Brazil; 3 Laboratory of Experimental Neurology, Graduate Program in Health Sciences, University of Southern Santa Catarina (UNESC), Criciúma, SC Brazil; 4 Pain Center, Anesthesiology Department, Washington University in Saint Louis, Fort Neuroscience Research Building, Medical Campus, St. Louis, MO 63110 USA

**Keywords:** Spent ground coffee oil, Chronic pain, Inflammation, Non-thermal plasma

## Abstract

Spent ground coffee oil (SGCO), rich in antioxidants and bioactive compounds, exhibits anti-inflammatory and analgesic properties and therefore warrants investigation in inflammatory pain models. This study aimed to investigate the topical effects of SGCO on hyperalgesia, emotional behavior, and inflammation-related mechanisms in a Complete Freund’s Adjuvant (CFA)-induced inflammation model. SGCO was extracted from spent coffee grounds via non-thermal plasma (NTP) and incorporated into a transdermal vehicle at 10%, 20%, and 30% (w/w). Female Balb/c mice (2 months old, *n* = 8/group) received daily topical applications of SGCO for 14 days after CFA-induced inflammation. Mechanical and cold nociception and paw edema were assessed. The concentration with the strongest antihyperalgesic effect (20%) was used in further experiments assessing mechanical conflict avoidance, anxiety- and depression-like behaviors, cytokine and oxidative parameters (paw, spinal cord, hippocampus, and prefrontal cortex), and inflammatory pathways in paw tissue. SGCO at 10%, 20%, and 30% produced significant antihyperalgesic effects without affecting paw edema, with 20% providing a sustained effect for 11 days. SGCO 20% was associated with reduced anxiety-like behavior, improved hedonic-like behavior without altering locomotion, and reduced pro-inflammatory cytokines (interleukin-1β and tumor necrosis factor), myeloperoxidase activity, phospho-Akt1/2/3, and NLRP3 in paw tissue. Additionally, it decreased protein carbonyl levels while increasing sulfhydryl group levels in the prefrontal cortex and hippocampus. These findings indicate that SGCO modulates inflammatory and oxidative pathways associated with CFA-induced inflammation and produces measurable effects on nociceptive and behavioral outcomes. Although the magnitude of the effects was generally modest, SGCO obtained via NTP technology may represent a low-cost and environmentally sustainable source of bioactive compounds for further preclinical investigation.

**Graphical Abstract:**

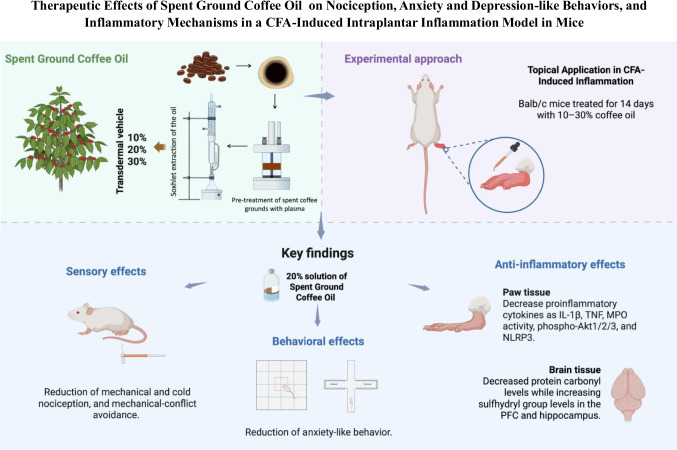

**Supplementary Information:**

The online version contains supplementary material available at 10.1007/s12035-026-06069-3.

## Introduction

Inflammatory arthritis constitutes a group of various rheumatic diseases characterized by inflammation of synovial joints and systemic manifestations [1]. These conditions are more prevalent in women than men and primarily affect adults aged 45 and older. When not managed properly, diseases of the joints and surrounding tissues, such as arthritis, can result in activity limitations, disability, and chronic pain [2]. Moreover, chronic pain is often accompanied by depression, anxiety, and stress, which contribute to emotional distress and further exacerbate the perception of pain [3, 4]. This landscape affects patients’ quality of life, reduces work productivity, and impairs long-term emotional health, making it a global public health concern [5, 6].

Inflammatory arthritis affects the entire joint structure [7] leading to symptoms such as movement-related joint pain, stiffness after periods of immobility, edema, deformity, instability, joint crepitus, and functional limitations in movement [8]. These symptoms result from a chronic inflammatory process in the joint tissues mediated by pro-inflammatory cytokines, such as tumor necrosis factor (TNF), interleukin (IL)−1β, IL-6, IL-8, IL-15, IL-17, and Interferon-γ (IFN-γ) [9, 10]. Persistent release of pro-inflammatory mediators hampers the resolution of the chronic inflammation process [11, 12], sensitizing central neurons within the nociceptive circuit and areas associated with pain perception, amplifying the peripheral painful signals [13]. While the integration and initial processing of sensory inputs occur in the spinal dorsal horn, the processing of sensory-discriminative, emotional-aversive, and experiential aspects of pain involves superior structures, such as the lateral and medial thalamus, limbic regions, and cortical areas [14].

Complete Freund’s Adjuvant (CFA) is an animal model widely used in literature that mimics human inflammatory arthritis. CFA stimulates the release of several inflammatory cytokines, such as IL-17, IL-1β, IL-6, TNF-α, IFN-γ, and high-sensitivity C-Reactive Protein (CRP) [15–18]. In addition, CFA stimulates reactive oxygen (ROS) and nitrogen species, leading to neutrophil infiltration and peripheral tissue damage [18]. CFA also up-regulates intracellular inflammatory pathways, including phospho-Akt [19], nuclear factor-kappa B (NF-κB), caspase-1, and NLRP3 inflammasome [17, 20]. These inflammatory mediators heighten pain sensitivity and trigger behavioral, emotional, and cognitive changes [21]. Thus, modulating inflammatory cytokines and ROS pathways has emerged as a potential therapeutic strategy for inflammatory pain management [18, 21].

In search of natural compounds capable of modulating these inflammatory pathways, coffee oil, particularly those derived from green and roasted beans, contains bioactive compounds such as chlorogenic acids (e.g., caffeic, ferulic, and quinic acids) and fatty acids (e.g., linoleic, oleic, palmitic, and stearic acids), which exhibit potent antioxidant and anti-inflammatory properties [22]. Topical application of these oils has shown to modulate cytokine secretion, accelerate wound healing, and produce systemic effects in animal models [22]. Given the widespread global consumption of coffee, estimated to generate over 6 million tons of spent coffee grounds annually, these residues pose a significant environmental burden due to their high volume and lack of commercial value [23, 24]. However, increasing attention has been directed toward the valorization of spent coffee grounds, which are rich in bioactive molecules with antioxidant and anti-inflammatory effects [25–27]. Recent studies have focused on the extraction of oil from spent coffee grounds, showing that this byproduct contains high levels of beneficial fatty acids and phenolic compounds [28]. Notably, the application of non-thermal plasma (NTP) as a pre-treatment enhances the oil extraction yield and improves its chemical profile, promoting a more sustainable and efficient recovery process [28]. NTP, a partially ionized gas generated by high-voltage discharge, induces electron avalanches and the formation of ionized chemical species and UV radiation, which play a role in enhancing extraction efficiency [29]. Altogether, these findings highlight the potential of spent coffee grounds oil as a low-cost, environmentally sustainable product with promising pharmaceutical applications.

In this study, we investigated the effects of spent ground coffee oil, extracted from spent coffee grounds pretreated with NTP, on mechanical hyperalgesia, nociception, edema, and emotional impairment induced by intraplantar (i.pl.) injection of Complete Freund’s Adjuvant (CFA) in mice. In addition, we evaluated the effects of spent ground coffee oil on peripheral and central mechanisms associated with CFA-induced inflammation, including cytokine levels, oxidative parameters, and intracellular inflammatory pathways.

## Material and Methods

### Animals

A total of 140 female Balb/c wild-type mice (*Mus musculus*), 2 months old, weighing between 25 and 30 g, were used. All animals were obtained from the Animal Facility of the Universidade do Extremo Sul Catarinense (UNESC). The number of animals per group was determined as previously described [30], using a 95% confidence interval: *N* = {[(z alpha + z beta) * s]/sigma}^2^. Thus, *N* = {[(1.96 + 1.28) * 35]/40}^2^ = 8. To account for inherent errors in biological experiments and potential sample loss, an additional 25% was added to the calculated sample size, resulting in a final group of 10 animals.

Animals were monitored daily for clinical signs of pain, distress, or illness (e.g., changes in posture, grooming, mobility, feeding, or hydration). Humane endpoints were predefined: animals exhibiting persistent weight loss greater than 20%, severe lethargy, inability to access food or water, or other signs of unrelieved distress would be removed from the study and humanely euthanized. No animals reached these predefined endpoints during the study. No specific inclusion or exclusion criteria were established a priori beyond the predetermined sample size calculation. All animals that received the planned treatments and completed the experimental timeline were included in the analyses, and no animals or data points were excluded during the experiment or data analysis.

The animals were housed and maintained in the animal facility of the Experimental Neuroscience Laboratory (LaNEx) of UNISUL at 22 ± 2 °C in a 12-h light/12-h dark cycle, with free access to food and water. All experimental procedures complied with the ethical principles for animal research established by the National Institutes of Health (NIH) and the Brazilian National Council for Control of Animal Experimentation (CONCEA). The study protocols were reviewed and approved by the Animal Use Ethics Committee of Universidade do Sul de Santa Catarina (protocol no 22.012.4.09.IV) and Universidade do Extremo Sul Catarinense (UNESC, protocol no 55/2022), and were designed, conducted, and reported in compliance with the ARRIVE guidelines [31].

### Experimental Design

**Fig. 1:**
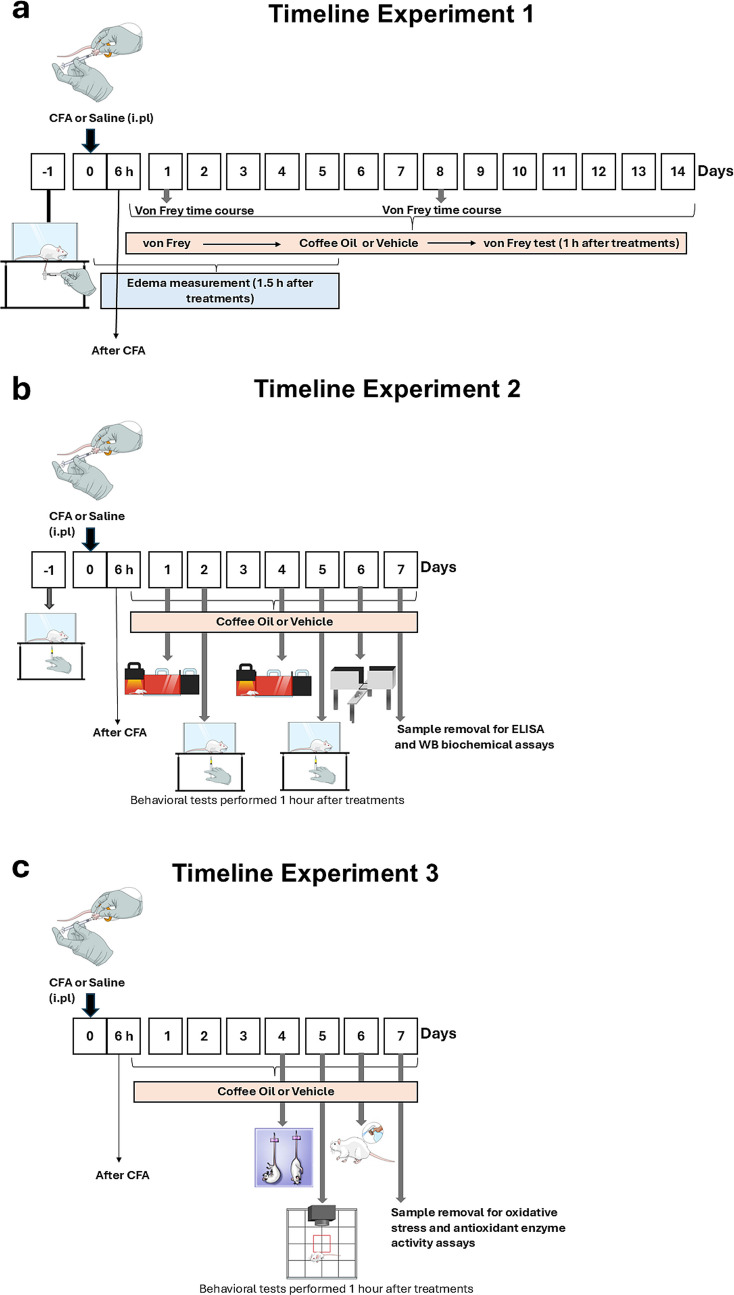
Overview of the experimental design. (**a**) Timeline of treatments and behavioral evaluations assessing the effects of Coffee Oil (10%, 20%, and 30%) on mechanical hyperalgesia and edema following CFA-induced inflammation. (**b**) Timeline of behavioral tasks used to evaluate the effects of Coffee Oil 20% on mechanical nociception and anxiety-like behavior after CFA-induced inflammation. (**c**) Timeline of behavioral assays used to assess depression-like behavior and locomotor activity after CFA-induced inflammation. CFA Complete Freund’s Adjuvant i.pl. intraplantar

The experimental design was divided into three experiments (Fig. 1). Animals were allocated to groups using a simple random draw without replacement. In experiment 1, animals were randomly distributed into six experimental groups (N = 10/group): (1) Saline/Vehicle; (2) Saline/Coffee Oil 20%; (3) CFA/Vehicle; (4) CFA/Coffee Oil 10%; (5) CFA/Coffee Oil 20%; and (6) CFA/Coffee Oil 30%. In this set of experiments, mechanical hyperalgesia was evaluated using the von Frey test (baseline, 6 h after CFA administration, and once a day until the 14th day after model induction) and edema measurement (baseline, 6 h after CFA administration, and once a day until the 5th day after model induction) were performed. The animals were treated with 10%, 20%, and 30% Coffee Oil or vehicle administered topically, 6 h after CFA administration and once a day until the 14th day (Fig. 1a).

In experiments 2 (Fig. 1b) and 3 (Fig. 1c), the animals were randomly divided into four groups (N = 10/group): (1) Saline/Vehicle; (2) Saline/Coffee Oil 20%; (3) CFA/Vehicle; and (4) CFA/Coffee Oil 20%. The animals were subjected to CFA or saline i.pl. and then treated with Coffee Oil 20% or vehicle administered topically six hours after CFA administration, and subsequently, once a day until the 7th day. In the second experiment animals were subjected to a Mechanical Conflict-Avoidance System (MCAS; Days 1 and 4), acetone test (Days 2 and 5), and elevated plus maze test (Day 6). On Day 7, the animals were euthanized for tissue collection for biochemical assays.

In the third experiment, following CFA-induced inflammation, the animals were subjected to the open field test (Day 5), the tail suspension test (Day 4), and the splash test (Day 6) to evaluate locomotion, depression- and anhedonic-like behaviors. On the 7th day, the animals were euthanized, and tissue was collected for biochemical assays to evaluate oxidative stress and antioxidant enzyme activity.

To minimize potential confounders, animals from different groups were tested in a randomized order across experimental days. Behavioral assessments were conducted at the same time of day (in the morning) to reduce circadian influences. All treatments were performed by the same trained experimenter to reduce inter-operator variability, as were the assessments. The allocation of animals to experimental groups was carried out in a blinded manner to ensure unbiased assignment. The personnel responsible for conducting the experiments, evaluating the outcomes, and analyzing the data were blinded to group allocation.

Additionally, an independent positive-control validation experiment using topical dexamethasone disodium phosphate (1 mg/g) was conducted to confirm the responsiveness of the CFA-induced inflammatory pain model to a standard anti-inflammatory intervention and to provide a pharmacological benchmark for contextualizing the magnitude of the effects observed with Coffee Oil 20%. Detailed methods and results are provided in Online Resource 1.

### CFA-Induced Inflammation Model

The inflammation model was induced by Complete Freund’s Adjuvant (F5881, Sigma-Aldrich, St. Louis, MO, USA), known to promote a persistent peripheral inflammatory response with peripheral and central sensitization effects [32]. Animals in the CFA/Vehicle and CFA/Coffee Oil groups were randomly selected from the housing box and received an i.pl. injection of CFA (20 μl dissolved in saline solution 70%) in the right hind paw. The Saline/Vehicle and Saline/Coffee Oil groups received an injection of 0.9% NaCl (20 μl, i.pl.) in the right hind paw.

### Spent Ground Coffee Oil Extraction for Topical Treatment

The coffee grounds samples were from Brazilian coffee, 100% Arabica (*Coffea arabica* L.) beans of controlled origin, supplied by a local coffee shop. Spent ground coffee oil was extracted using a non-thermal plasma (NTP) pretreatment followed by Soxhlet extraction. Briefly, coffee grounds were dried in an oven at 50 °C, then 10 g of coffee grounds were diluted in 50 mL of distilled water and treated in an NTP reactor for 10 min under stirring. The non-thermal plasma reactor with quartz walls and Teflon caps, coupled to a power source of 17 kV and 30 mA of AC current, was used to generate the plasma using tip-flat electrodes operating at atmospheric pressure under argon gas of 3 L min − 1. The treated grounds were placed back in the oven and dried at 50 °C. Finally, 100 g of grounds with 300 mL of N-Hexane were used for oil extraction using the Soxhlet method, which allows solvent reuse without releasing compounds into the environment [28].

### Topical Treatment with Coffee Oil Extracted from Coffee Grounds on the CFA-Induced Inflammation Model

The oil extracted from coffee grounds was incorporated into Pentravan® (Fagron, São Paulo, Brazil), a commercially available liposomal transdermal vehicle composed of purified water, phosphatidylcholine (lecithin), isopropyl myristate, propylene glycol, fatty alcohols, and preservatives. This vehicle is widely used to enhance skin permeation and enable the transdermal delivery of drugs into systemic circulation. The oil was added at concentrations of 10%, 20%, and 30% (w/w). This technology is commonly employed in topical formulations when systemic effects are desired beyond the local site of application.

Topical treatment was applied to the right hind paw (80 mg/paw) beginning 6 h after CFA or saline injection (Day 0) and was subsequently administered once daily at the same hour each morning (approximately 9:00 a.m.) to ensure consistent timing and minimize circadian variability. Behavioral tests were performed 1 h after treatment, corresponding to the expected time for transdermal absorption and onset of pharmacodynamic effects, whereas paw-edema measurements were performed 1.5 h after treatment on Days 0–5. Animals in the Saline/Vehicle and CFA/Vehicle groups were treated according to the same schedule using the oil-free transdermal vehicle.

### Mechanical Hyperalgesia

Mechanical hyperalgesia assessments were randomly performed between groups using 0.6 g von Frey monofilaments (VFH, Stoelting, IL, USA). Each animal was placed individually in bottomless acrylic chambers with a lid (9 cm × 7 cm × 11 cm) over a wire mesh, allowing the monofilament to be applied perpendicularly to the plantar surface of the paw. The curvature of the filament indicated sufficient pressure, and paw withdrawal was recorded as a positive nociceptive response to 10 filament applications, expressed as a response percentage [33]. Mechanical hyperalgesia was used as the primary outcome measure, as it reflects the core objective of assessing nociceptive sensitivity in the inflammatory pain model.

### Thermal Allodynia

Cold allodynia was assessed using the acetone test. The animals were placed individually in an acrylic box (9 cm × 7 cm × 11 cm) without a bottom and covered with a lid, positioned over a wire mesh to allow access to the plantar surface of the hind paw. A syringe was used to squirt 20 µL of acetone to the right hind paw, and pain behaviors such as paw withdrawal, shaking, and licking in response to the cooling were observed over 35 s. The response time (in seconds) after acetone application was measured [34].

### Measurement of Paw Edema

Paw edema was assessed with a universal digital micrometer [30]. Edema was expressed by measuring the thickness from the back to the plantar part of the right paw of each animal.

### Mechanical Conflict-Avoidance System

The test assesses the cognitive component of pain processing using an un-evoked behavioral assessment of pain. The apparatus with three transparent red acrylic chambers was used. Chamber 1, illuminated with a light-emitting diode (LED), serves as a slightly aversive stimulus, encouraging the animal to “escape” to the dark chambers. This chamber is separated from the others by a movable barrier. Chamber 2, the test chamber, connects chambers 1 and 3. Its floor consists of steel probes (0.5 mm in diameter), set at 2 mm and 5 mm heights to generate a mechanical stimulus to the animal’s paws. The animals were placed in chamber 1 with the barrier closed for the test. The LED was turned on after 15 s, and the barrier was removed 20 s later. The latency to leave the light compartment (with all four paws) once the door was opened, as well as the time taken to reach the dark compartment with all four paws, were recorded in seconds and expressed in the graph as escape latency. The increase in latency indicates mechanical conflict avoidance, characterizing mechanical hypersensitivity [35].

### Elevated Plus Maze Test

The test evaluates the natural tendency of mice to explore a new environment and the aversive properties of an open arena. The acrylic apparatus consists of two open arms (W 6 cm × L 35 cm, without walls) and two closed arms (W 6 cm × L 35 cm, flanked by 17 cm dark walls), which extend from a common central zone, located 70 cm above the floor.

The animals were placed in the central zone facing an open arm, allowing them to freely explore each space of the apparatus for 5 min. The number of entries and the total time in the open arms (in seconds) were measured and displayed in the graphs [36].

### Open Field Test

The open-field test was used to evaluate both locomotor activity and anxiety-like behavior (thigmotaxis) [37]. The apparatus consisted of a matte light gray wooden square box measuring 40 cm × 40 cm × 40 cm. Each animal was individually placed in the center of the arena and allowed to freely explore for 5 min. Locomotor activity was quantified by the total distance traveled (meters), while anxiety-like behavior was assessed by the number of entries into the center and the time spent in the center of the arena (seconds). These parameters were automatically recorded and analyzed using ANYmaze® software (Stoelting, USA). The apparatus was cleaned with a 10% ethanol solution between sessions to eliminate olfactory cues from previous animals [38].

### Tail Suspension Test

The mice were suspended by the tail 50 cm above ground using adhesive tape, and the immobility time (in seconds) was recorded over 5 min. An increase in the duration of immobility was interpreted as an indicator of a depressogenic effect [39].

### Splash Test

Spraying a 10% sucrose solution on the dorsal coat induces grooming behavior, indicating self-care and motivational behavior. The total time (in seconds) the animal spent engaged in self-grooming was timed over 5 min [40].

### Biochemical Assays

Biochemical assays were performed seven days after the administration of CFA or saline and 1 h after the last treatment with Coffee Oil 20% or vehicle. The animals were euthanized by decapitation (1–2% isoflurane with 100% oxygen) for the removal of the right hind paw (skin, muscles, and joints), lumbar spinal cord (L4-L6), PFC, and hippocampus. Immediately after dissection, the samples were frozen in liquid nitrogen and stored in a − 80 °C freezer until analyses were conducted.

### Enzyme-Linked Immunosorbent Assay (ELISA)

The samples were homogenized using an Ultra-Turrax Homogenizer (T-18, IKA Works, Wilmington, NC, USA) in phosphate-buffered saline (PBS) containing Tween 20 (0.05%), PMSF (0.1 mM), EDTA (10 mM), aprotinin (2 ng/ml), and benzethonium chloride (0.1 mM). The samples were then centrifuged at 12,000 × g for 15 min (4 °C), and the supernatant was collected and stored at − 80 °C until analyses. Protein quantification was determined using the Bradford method [41]. Aliquots of 100 μl of the supernatant were used to measure the concentrations of cytokines TNF (Cat. DY210, Assay Range: 15.6–1000 pg/mL), IL-1β (Cat. DY201, Assay Range: 3.9–250 pg/mL), IL-6 (Cat. DY206, Assay Range: 9.4–600 pg/mL), IL-10 (Cat. DY217B, Assay Range: 31.2–2000 pg/mL), IL-17 (Cat. DY317, Assay Range: 15.6–1000 pg/mL), IFN-γ (Cat. DY285B, Assay Range: 9.4–600 pg/mL) and CRP (Cat. DY1829, Assay Range: 23.4–1500 pg/mL), using mouse DuoSet ELISA kits (R&D Systems, Minneapolis, USA), according to the manufacturer’s instructions, through a colorimetric assay.

Cytokine concentrations were determined by interpolating absorbance data with a 7-point standard curve, measured at 450 nm (with 540-nm wavelength correction) using an ELISA plate reader (Perlong DNM-9602, Nanjing Perlove Medical Equipment Co., Nanjing, China). Results were expressed in pg/mg of protein [42].

### Western Blotting Analyses

The Western blot assay was used to quantify the immunocontent of intracellular inflammatory pathways NLRP3 inflammasome and Akt1/2/3 phosphorylation. Samples were homogenized and incubated in RIPA lysis buffer with a 1% protease inhibitor cocktail, centrifuged at 10,000 × g for 10 min (4 °C). The supernatant was collected and diluted in electrophoresis buffer. Protein content was measured using the Bradford method. Samples were heated at 95 °C for 5 min and stored in a − 80 °C freezer.

Proteins (50 μg) electrophoresis were performed using a 10% SDS–polyacrylamide running gel. Proteins were then transferred to a nitrocellulose membrane, blocked, and incubated (4 °C) with primary antibodies: anti-NLRP3 (Invitrogen, PA5-115660, 1:1000) and anti-phospho-Akt1/2/3 (Santa Cruz Biotechnology, sc-33437, 1:500). After washing, membranes were incubated with the appropriate peroxidase-conjugated secondary antibody at room temperature for one hour (1:10,000). Membranes were then exposed to a chemiluminescent detection kit and visualized using an imaging system (iBright Imaging Systems, Invitrogen/Thermo Fisher Scientific, Waltham, MA, USA). Quantitative analysis of the bands was performed by densitometry using the imaging system software. Values were normalized using β-actin (Sigma-Aldrich, A3854, 1:50,000) values and expressed graphically as arbitrary units relative to the control group.

### Colorimetric Assays for Oxidative Stress and Antioxidant Enzyme Activity

Oxidative damage to proteins was quantified by measuring carbonyl group levels through reaction with dinitrophenylhydrazine (Sigma-Aldrich). Briefly, proteins were precipitated with 20% trichloroacetic acid (Vetec) and redissolved in dinitrophenylhydrazine (Sigma-Aldrich). Absorbance was measured at 340 nm using a spectrophotometer, and results were expressed in nmol/mg of protein [13].

Lipid damage was quantified by measuring thiobarbituric acid-reactive substances (TBARS) levels. Briefly, samples were homogenized in phosphate buffer (Nuclear) and deproteinized with 10% trichloroacetic acid (Vetec), followed by centrifugation at 3000 rpm for 10 min. The reaction was initiated by adding 0.67% thiobarbituric acid to the supernatant and heating it to 100 °C for 30 min. Absorbance was measured at 532 nm using a spectrophotometer, with 1,1,3,3-tetramethoxypropane (Sigma-Aldrich) as an external standard. Results were expressed in nmol of malondialdehyde equivalents per mg of protein [43].

Myeloperoxidase (MPO) was measured as an indicator of neutrophil infiltration. Briefly, structures were homogenized in 0.5% hexadecyltrimethylammonium bromide (Sigma-Aldrich) and centrifuged at 15,000 rpm for 15 min. A 1.6 mM tetramethylbenzidine solution (Labdel) was added to the supernatant and incubated for 5 min at 37 °C, followed by the addition of 1 mM H₂O₂ and further incubation at 37 °C for 30 min. Absorbance was measured at 650 nm using a spectrophotometer. Results were expressed in U/mg of protein [44].

The concentration of nitrite/nitrate was determined using the Griess reaction. Briefly, the reaction was carried out by adding the Griess reagent (0.1% N-(1-Naphthyl)ethylenediamine dihydrochloride and 1% sulfanilamide in a 1:1 ratio; Synth) and vanadium (III) chloride (Sigma-Aldrich) to the tissue previously homogenized in phosphate buffer (Nuclear). After 1 h of incubation at room temperature, protected from light, absorbance was measured at 540 nm using a spectrophotometer. Results were expressed in nmol/mg of protein [45].

Catalase (CAT) enzyme activity was determined by measuring the decrease in hydrogen peroxide consumption. Tissue samples were homogenized in 1 mL of catalase buffer [PBS + NaCl (pH 7.0), containing NaCl (136.9 mM), KH₂PO₄ (1.1 mM), and Na₂HPO₄ (0.27 mM)] and centrifuged for 10 min at 3000 rpm at room temperature. A 100 µL sample supernatant was mixed with 1 mL of catalase buffer containing H₂O₂ (prepared by adding 400 µL of H₂O₂ to 25 mL of catalase buffer, protected from light). Absorbance readings were taken at 240 nm using a deuterium lamp spectrophotometer in quartz cuvettes, with measurements at 0, 30, and 60 s. The process was repeated for each sample, and CAT activity was expressed in U of CAT/mg of protein [46].

Oxidative damage to proteins in the sulfhydryl group was assessed by measuring sulfhydryl groups using dithiobis nitrobenzoic acid (DTNB). Samples were precipitated, and proteins were dissolved in a DTNB solution. After 30 min of incubation at room temperature, sulfhydryl groups were measured by absorbance at 412 nm. Results were expressed in nmol/mg of protein [47].

### Statistical Analyses

The results were analyzed using the GraphPad Prism program, version 9.0 (La Jolla, California, USA). The normality of the data was verified by the Shapiro–Wilk test, being considered parametric. Data were expressed as mean ± standard deviation (SD). Because unforced behavioral paradigms in female Balb/c mice naturally show greater inter-individual variability, this variation was expected across several behavioral outcomes and does not compromise the validity of the statistical comparisons. Comparison between groups was performed using two-way analysis of variance (ANOVA), followed by the Tukey test. This post hoc procedure was selected because it provides adjusted *p*-values that account for multiple pairwise comparisons and effectively controls the family-wise type I error rate.

Effect size analyses were conducted to quantify the magnitude of the effects of Coffee Oil treatments when statistically significant differences were detected in the Tukey post hoc test. In these cases, effect sizes were calculated based on point-by-point simple-effects comparisons between Coffee Oil–treated groups and the CFA/Vehicle group. For von Frey assessments involving daily evaluation and time-course analyses, effect sizes were calculated using area under the curve (AUC) analysis to quantify the overall magnitude of the treatment effect across time. In the von Frey time-course experiments, AUC calculations were restricted to the first 3 h after treatment, corresponding to the time window in which significant post hoc differences were observed. Effect sizes were estimated using Hedges’ g and reported with 95% confidence intervals. Magnitudes were interpreted according to conventional thresholds, with values of approximately 0.2 indicating small effects, 0.5 moderate effects, and values ≥ 0.8 indicating large effects. For all Hedges’ g analyses, effect sizes were calculated relative to the CFA/Vehicle group. Negative values indicate lower outcomes in the treatment group compared with CFA/Vehicle, whereas positive values indicate higher outcomes in the treatment group compared with CFA/Vehicle. *P* values less than 0.05 were considered statistically significant in all analyses.

## Results

### Topical Treatment with Spent Ground Coffee Oil 20% Demonstrated Prolonged Antihyperalgesic Effect in a CFA-Induced Inflammation Model

**Fig. 2:**
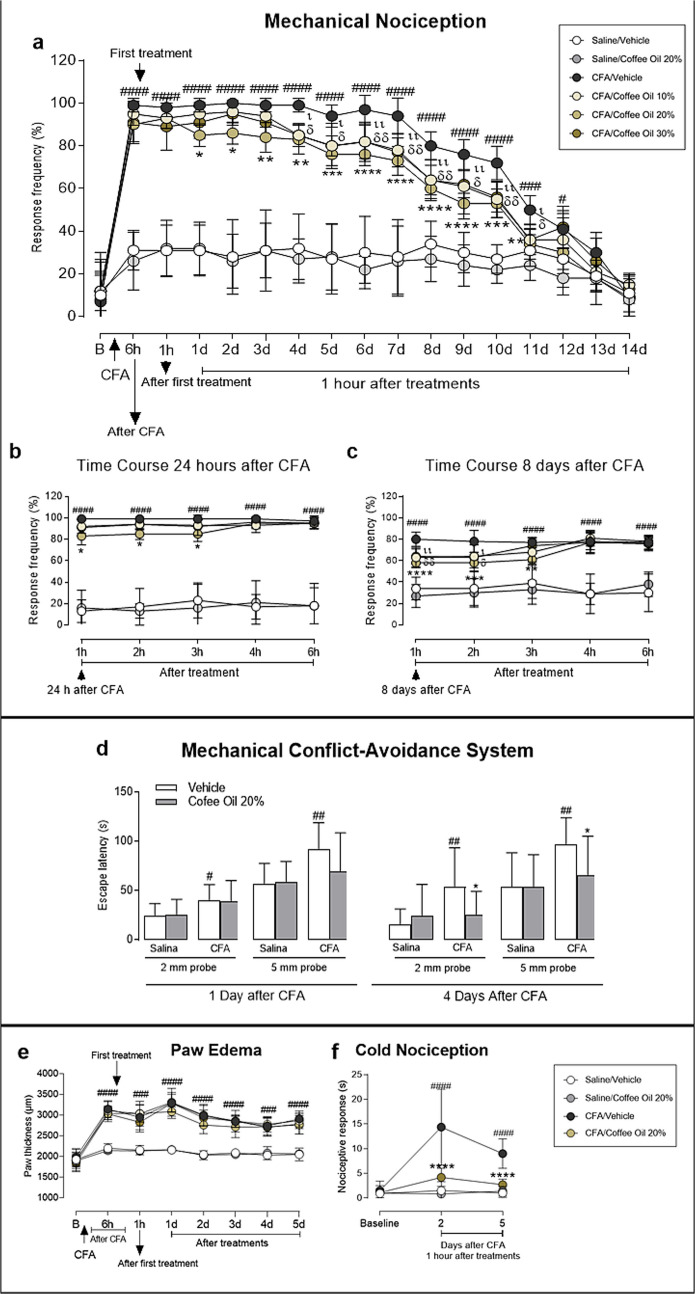
Effects of Coffee Oil treatment at 10%, 20%, and 30% on mechanical nociception and paw edema after CFA-induced inflammation. Basal mechanical stimulus response frequency (a left), six hours (**a**), and 1 to 14 days (a) after CFA and after first and consecutive treatments (a right). Time course of mechanical hyperalgesia at 24 h (**b**) and 8 days (**c**) post-CFA. Mechanical nociception evaluated by the mechanical conflict-avoidance system 24 h and 4 days after CFA with Coffee Oil 20% treatment (**d**). Time course of paw edema (**e**) and cold nociception (f) after CFA and Coffee Oil treatments. Values represent mean ± SD (*n* = 10 animals/group). Two-way ANOVA with repeated measures followed by Tukey’s post hoc test. # *p* < 0.05, ## *p* < 0.01, ### *p* < 0.001, #### *p* < 0.0001 compared with Saline/Vehicle for Sham/Vehicle. * *p* < 0.05, ** *p* < 0.01, *** *p* < 0.001, **** *p* < 0.0001 compared with CFA/Vehicle for CFA/Coffee Oil 20%. ι *p* < 0.05, ιι *p* < 0.01, ιιι *p* < 0.001 compared with CFA/Vehicle for CFA/Coffee Oil 10%. δ *p* < 0.05, δδ *p* < 0.01 compared with CFA/Vehicle for CFA/Coffee Oil 30%. CFA Complete Freund’s Adjuvant

The first experimental design (Fig. 1A) evaluated the effect of Coffee Oil 10%, 20%, and 30% on mechanical hyperalgesia and paw edema after CFA-induced inflammation. Intraplantar administration of CFA induces persistent hyperalgesia, increasing the responsiveness to mechanical stimulus, from 6 h to 12 days after administration. The topical treatment with Coffee Oil 20% demonstrated an antihyperalgesic effect, reducing the response frequency from the first day up to the 11th day after CFA-induced inflammation. The treatment with Coffee Oil 10% and 30% presented an antihyperalgesic effect from four up to 11th days after CFA-induced inflammation, as indicated by a reduction in the response frequency of CFA/Coffee Oil groups (Fig. 2a). AUC analysis was used to calculate effect sizes and revealed significant differences for CFA/Coffee Oil 10% (Hedges’ g =  − 3.42; 95% CI: − 4.80 to − 2.01), CFA/Coffee Oil 20% (Hedges’ g =  − 5.69; 95% CI: − 7.70 to − 3.65), and CFA/Coffee Oil 30% (Hedges’ g =  − 4.90; 95% CI: − 6.68 to − 3.09) compared with CFA/Vehicle. Comparisons between doses showed that Coffee Oil 20% differed significantly from both Coffee Oil 10% (Hedges’ g = 1.56; 95% CI: 0.57 to 2.53) and Coffee Oil 30% (Hedges’ g =  − 1.81; 95% CI: − 2.81 to − 0.77) in the AUC analysis, whereas no significant difference was observed between the 10% and 30% formulations (Hedges’ g = 0.11; 95% CI: − 0.73 to 0.95) (Fig. 2a).

The time course of response frequency indicated Coffee Oil 20% exhibited an antihyperalgesic effect up to three hours after treatment, both 24 h (Fig. 2b) and 8 days (Fig. 2c) after CFA administration. AUC analysis restricted to the first 3 h after treatment, corresponding to the time window in which significant post hoc differences were detected, showed that Coffee Oil 20% differed from CFA/Vehicle at both 24 h (Hedges’ g =  − 3.73; 95% CI: − 5.18 to − 2.24) and 8 days after CFA administration (Hedges’ g =  − 3.26; 95% CI: − 4.59 to − 1.89). Coffee Oil 10% and 30% reduced the response frequency for up to 2 h on day 8 (Fig. 2c), with no effect during the first 24 h after CFA-induced inflammation (Fig. 2b). Accordingly, at 8 days after CFA administration, AUC analysis revealed significant differences between CFA/Vehicle and both Coffee Oil 10% (Hedges’ g =  − 1.61; 95% CI: − 2.59 to − 0.61) and Coffee Oil 30% (Hedges’ g =  − 2.22; 95% CI: − 3.31 to − 1.10). Therefore, Coffee Oil 20% was selected for the subsequent experiments.

### Topical Treatment with Spent Ground Coffee Oil 20% Reduced Mechanical Hypersensitivity Conflict in a CFA-Induced Inflammation Model

Our results indicate that CFA administration induces mechanical hypersensitivity, as reflected by increased escape latency in the mechanical conflict-avoidance system on Day 1 and Day 4 after intraplantar injection (for both 2-mm and 5-mm probe conditions) (Fig. 2d). On the first day after CFA injection, treatment with Coffee Oil 20% did not alter the mechanical conflict-avoidance induced by CFA. On Day 4 after CFA administration, Coffee Oil 20% reduced escape latency in the mechanical conflict-avoidance system compared with CFA/Vehicle animals for both probe conditions (2-mm probe: Hedges’ g =  − 0.84; 95% CI: − 1.61 to − 0.23; 5-mm probe: Hedges’ g =  − 0.88; 95% CI: − 1.71 to − 0.25) (Fig. 2d).

### Topical Treatment with Spent Ground Coffee Oil did not Change Edema in a CFA-Induced Inflammation Model

CFA induces persistent paw edema, as evidenced by increased paw thickness from 6 h to 5 days after i.pl. administration. Treatment with 10%, 20%, or 30% of Coffee Oil did not affect the edema caused by the CFA (Fig. 2e).

### Topical Treatment with Spent Ground Coffee Oil 20% Exhibited Cold Antinociception Effect in a CFA-Induced Inflammation Model

Topical treatment with Coffee Oil 20% significantly reduced CFA-induced cold allodynia at both 2 and 5 days after intraplantar CFA injection (Fig. 2f). At Day 2 post-CFA, treatment with Coffee Oil 20% attenuated CFA-induced cold hypersensitivity compared with CFA/Vehicle animals, as indicated by a marked difference between groups (Hedges’ g =  − 1.51; 95% CI: − 2.39 to − 0.99), without relevant effects in saline-treated animals. This antinociceptive effect was sustained at Day 5, again showing a robust reduction of cold allodynia in the CFA/Coffee Oil 20% group (Hedges’ g =  − 2.74; 95% CI: − 4.15 to − 2.06).

### Topical Treatment with Spent Ground Coffee Oil 20% Exhibited an Anxiolytic Effect in a CFA-Induced Inflammation Model

**Fig. 3:**
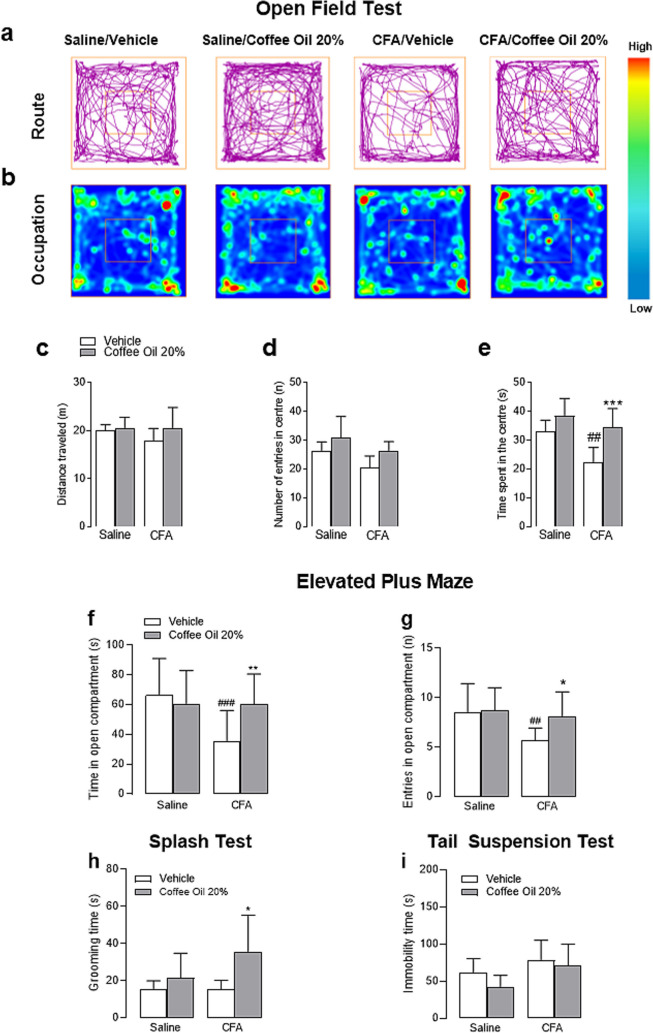
Effects of Coffee Oil 20% on locomotor activity anxiety-like, hedonic-like, and depression-like behaviors after CFA-induced inflammation. Track plot (**a**), occupancy (**b**), distance traveled (**c**), entries into the center (**d**), and time spent in the center (**e**) of the open field test. Time (**f**) and entries (**g**) into the open arms of the plus-maze test. Grooming (**h**) and immobility time in the splash and tail suspension tests respectively. Values represent mean ± SD (*n* = 10 animals/group). Two-way ANOVA followed by Tukey’s post hoc test. ## *p* < 0.01 compared with Saline/Vehicle. * *p* < 0.05, *** *p* < 0.001 compared with CFA/Vehicle. CFA Complete Freund’s Adjuvant

CFA administration did not alter spontaneous locomotor activity, as indicated by similar total distance traveled (Fig. 3c), track plots (Fig. 3a), and general occupancy patterns (Fig. 3b) in the open-field test. Importantly, despite preserved locomotion, CFA induced an anxiety-like profile characterized by increased thigmotaxis, evidenced by a reduced number of entries into the center (Fig. 3d) and reduced time spent in the center of the arena (Fig. 3e) in the open-field test, as well as decreased time spent and entries in the open arms of the elevated plus maze (Fig. 3F–G). Treatment with Coffee Oil 20% restored the anxiety-like behavior induced by CFA, as evidenced by increased exploration of anxiogenic zones, including greater time spent and number of entries in the open arms of the elevated plus maze (Hedges’ g for time = 1.19; 95% CI: 0.52 to 2.18; Hedges’ g for entries = 1.21; 95% CI: 0.71 to 1.90), as well as increased time spent in the center of the arena in the open field test (Hedges’ g = 1.95; 95% CI: 0.76 to 3.14) (Fig. 3e–g). CFA did not alter hedonic- and depression-like behavior assessed in the splash and tail suspension test, respectively (Fig. 3h, i). Coffee Oil 20% increases hedonic-like behavior, as evidenced by an increase in the grooming time of the CFA/Coffee Oil group (Hedges’ g = 1.33; 95% CI: 0.83 to 2.62; Fig. 3h).

### Spent Ground Coffee Oil 20% Reduced Pro-inflammatory Cytokine Levels and Suppressed p-Akt1/2/3 and NLRP3 Activation Peripherally in a CFA-Induced Inflammation Model

**Fig. 4:**
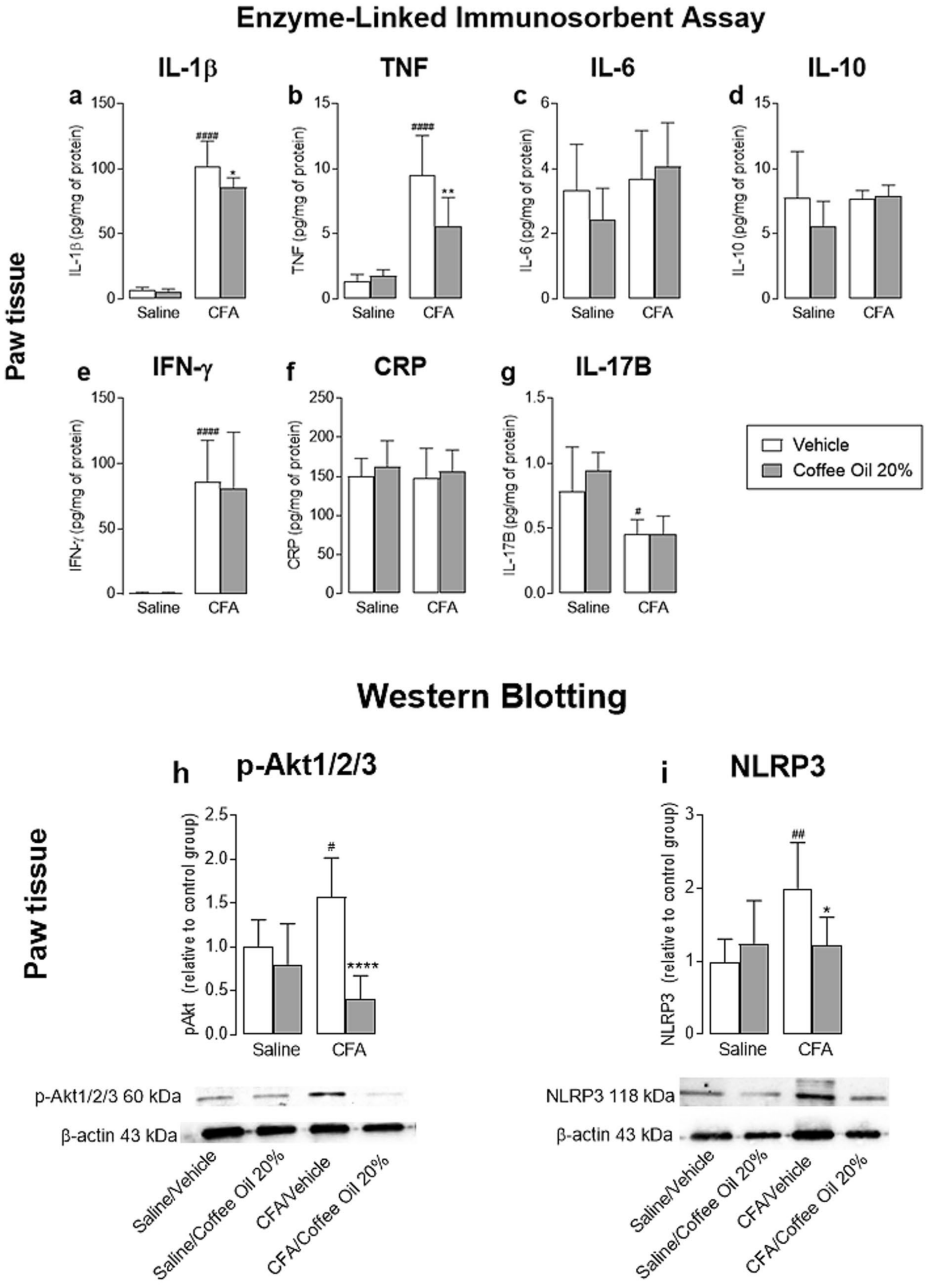
Effects of Coffee Oil 20% treatment on anti- and pro-inflammatory cytokine levels in paw tissue after CFA-induced inflammation. Levels of IL-1β (**a**), TNF (**b**), IL-6 (**c**), IL-10 (**d**), IFN-γ (**e**), CRP (**f**), IL-17B (**g**), p-Akt1/2/3 (**h**), and NLRP3 (**i**). Values represent mean ± SD (*n* = 10 animals/group). Two-way ANOVA followed by Tukey’s post hoc test. # *p* < 0.05, ## *p* < 0.01, #### *p* < 0.0001 compared with Saline/Vehicle. * *p* < 0.05, ** *p* < 0.01, **** *p* < 0.0001 compared with CFA/Vehicle. CFA Complete Freund’s Adjuvant

CFA administration induces peripheral inflammation, as indicated by increased levels of pro-inflammatory cytokines IL-1β, TNF, IFN-γ, and reduced levels of IL-17B in paw tissues (Fig. 4a, b, e, g). Coffee Oil 20% attenuates the levels of IL-1β (Hedges’ g =  − 0.99; 95% CI: − 3.84 to − 0.06) and TNF (Hedges’ g =  − 1.35; 95% CI: − 2.43 to − 0.70) increased by CFA (Fig. 4a, b) without affecting the remaining cytokines. The administration of CFA also induces intracellular alterations in the inflammatory pathways, such as an increase in the immunocontent of p-Akt1/2/3 and NLRP3, observed in the paw tissue. Our findings demonstrate that treatment with Coffee Oil 20% suppresses both p-Akt1/2/3 (Hedges’ g =  − 2.96; 95% CI: − 5.90 to − 2.09) and NLRP3 (Hedges’ g =  − 1.42; 95% CI: − 2.53 to − 0.83) levels increased by CFA (Fig. 4h, i).

### Spent Ground Coffee Oil 20% Up-regulated Pro- and Anti-inflammatory Cytokine Levels in the Hippocampus in a CFA-Induced Inflammation Model

**Fig. 5:**
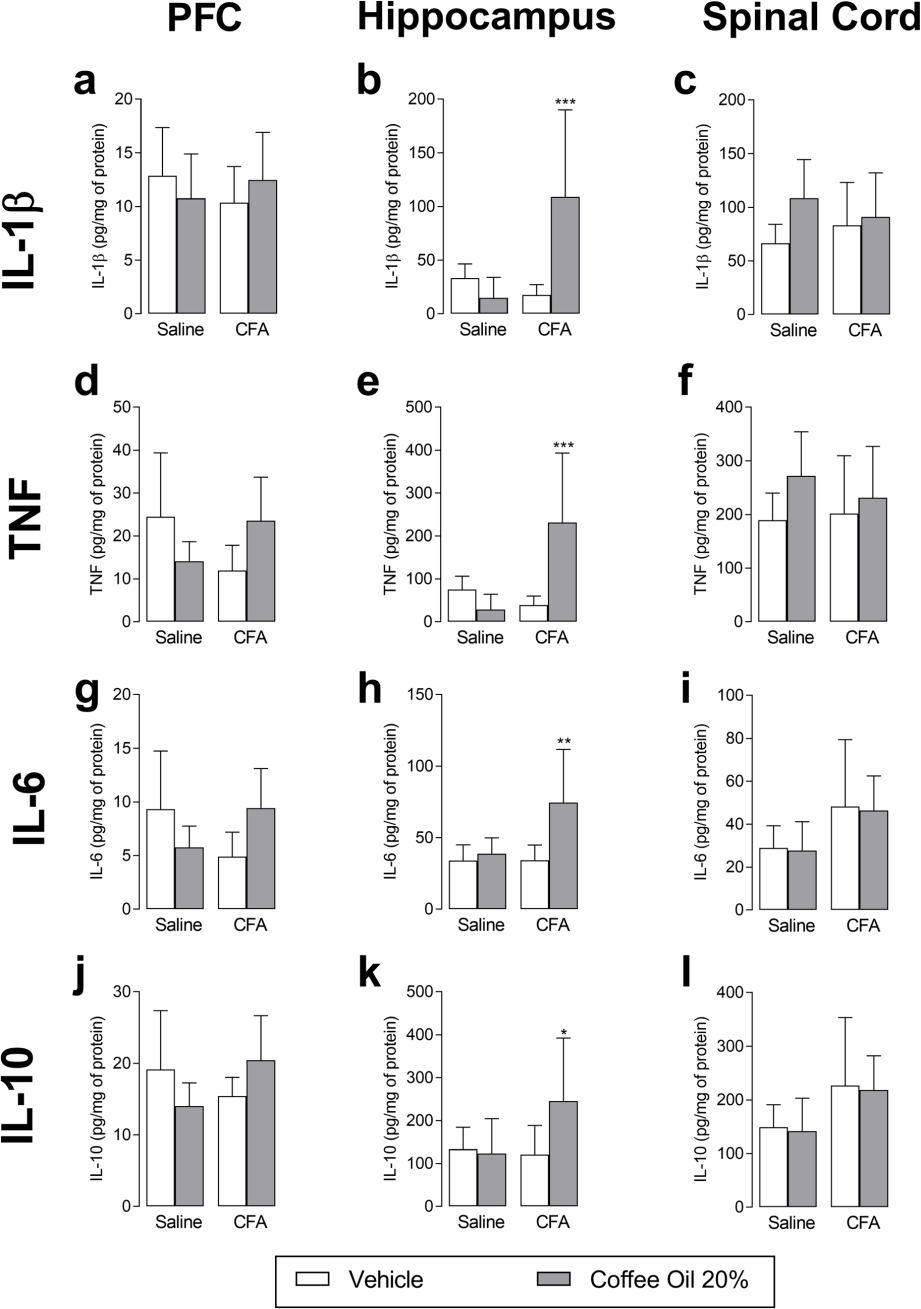
Effects of Coffee Oil 20% on cytokine levels in prefrontal cortex (PFC) (**a**, **d**, **g**, **j**), hippocampus (**b**, **e**, **h**, **k**), and spinal cord (**c**, **f**, **i**, **l**) after CFA-induced inflammation. Levels of IL-1β (a–c), TNF (d–f), IL-6 (g–i), and IL-10 (j–l). Values represent mean ± SD (*n* = 10 animals/group). Two-way ANOVA followed by Tukey’s post hoc test. ## *p* < 0.01 compared with Saline/Vehicle. * *p* < 0.05, *** *p* < 0.001 compared with CFA/Vehicle. CFA Complete Freund’s Adjuvant

CFA did not alter cytokine levels in the central nervous system (CNS), including PFC, hippocampus, and spinal cord (Fig. 5a–l). In contrast, Coffee Oil 20% treatment increased hippocampal cytokine levels in CFA-treated animals. Specifically, post hoc analysis revealed higher levels of IL-1β (Hedges’ g = 1.51; 95% CI: 1.01 to 2.86), TNF (Hedges’ g = 1.59; 95% CI: 1.06 to 2.97), IL-6 (Hedges’ g = 1.36; 95% CI: 0.66 to 2.79), and IL-10 (Hedges’ g = 1.03; 95% CI: 0.41 to 1.94) in the hippocampus of the CFA/Coffee Oil 20% group compared with the CFA/Vehicle group (Fig. 5b, e, h, k).

### Spent Ground Coffee Oil 20% Decreased Protein Carbonyls and Myeloperoxidase Levels in the PFC and Paw in a CFA-Induced Inflammation Model

**Fig. 6:**
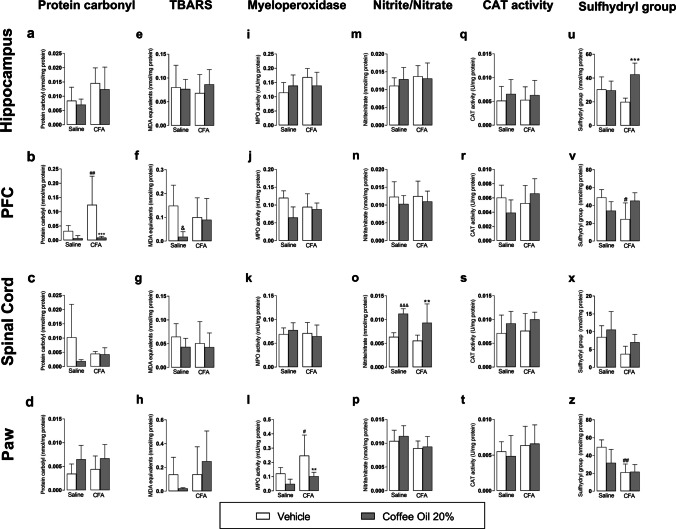
Effects of Coffee Oil 20% on oxidative stress markers in hippocampus (a, e, i, m, q, u), PFC (b, f, j, n, r, v), spinal cord (c, g, k, o, s, x), and paw tissue (d, h, l, p, t, z) after CFA-induced inflammation. Levels of protein carbonyls (**a**–**d**), TBARS (**e**–**h**), myeloperoxidase (**i**–**l**), nitrite/nitrate (**m**–**p**), catalase activity (**q**–**t**), and sulfhydryl groups (**u**–**z**). Values represent mean ± SD (*n* = 10 animals/group). Two-way ANOVA followed by Tukey’s post hoc test. # *p* < 0.05, ## *p* < 0.01 compared with Saline/Vehicle. & *p* < 0.05 compared with Saline/Vehicle. ** *p* < 0.01, **** *p* < 0.0001 compared with CFA/Vehicle. CAT catalase CFA Complete Freund’s Adjuvant

CFA administration increases protein carbonyl levels and decreases sulfhydryl group level (Fig. 6b, v) in the PFC. CFA also increases myeloperoxidase levels and decreases the sulfhydryl group in the paw tissue (Fig. 6l, z). Treatment with Coffee Oil 20% partially mitigates the effects induced by CFA, normalizing protein carbonyls in the PFC (Hedges’ g =  − 1.66; 95% CI: − 4.94 to − 0.88) and myeloperoxidase levels in the paw (Hedges’ g =  − 1.32; 95% CI: − 2.55 to − 0.81). Coffee Oil 20% treatment also increases sulfhydryl groups in the hippocampus (Hedges’ g = 2.96; 95% CI: 2.37 to 5.71) and nitrite/nitrate levels in the spinal cord (Hedges’ g = 1.19; 95% CI: 0.45 to 2.89) (Fig. 6).

## Discussion

In this study, we investigated the effects of topical treatment with Spent Ground Coffee Oil on nociception, as well as inflammation‑related mechanisms in a CFA‑induced inflammation model in mice. Our results revealed that topical treatment with Spent Ground Coffee Oil attenuated mechanical and cold hypersensitivity, reduced mechanical conflict avoidance, and was associated with improvements in selected behavioral outcomes related to pain processing. These outcomes were supported by statistically significant differences and by effect-size estimates, which generally indicated effects ranging from moderate to large in magnitude across several behavioral and biochemical measures. Taken together, these results suggest that Coffee Oil exerts a biologically measurable, although not universal, modulation of the pain phenotype. Coffee Oil 20% incorporated into the transdermal vehicle modulated peripheral and central inflammatory mechanisms, reducing pro‑inflammatory cytokines and intracellular inflammatory pathways activated by CFA in the paw tissue, while exhibiting antioxidant effects both centrally and peripherally. These biochemical changes were accompanied by effect-size estimates that, in several outcomes, reached moderate-to-large magnitude, supporting the functional relevance of these modulatory actions. Interestingly, the intermediate concentration (20%) produced the most consistent and prolonged antihyperalgesic effect, suggesting that the dose–response relationship for Coffee Oil may not be strictly linear, in line with reports describing non‑monotonic patterns where intermediate doses can be more effective than either lower or higher doses [48, 49].

The present study also highlights the role of Coffee Oil extracted by non-thermal plasma, a technique that allows the reuse of coffee residue, reducing environmental impact, in a CFA-induced inflammation model in mice. Coffee Oil extracts have been known for their antioxidants, anti-inflammatory, and immunomodulator agents [26, 28] due to large amounts of linoleic acid, palmitic acid, oleic acid, stearic acid, arachidic acid, gadoleic acid, linolenic acid and behenic acid, eicosanoic and tricosanoic acid, total phenolics such as chlorogenic acids and caffeine [26, 28, 44, 50]. Previous studies identified 26 metabolites with known anti-inflammatory activities in spent coffee ground extracts [26]. This is a pioneering study evaluating the effects of oil extracted from spent coffee grounds in animal models, highlighting their potential anti-inflammatory role. Given this diverse chemical profile, it is plausible that the observed effects arise from selective modulation of intracellular inflammatory pathways rather than a generalized anti-inflammatory action, which aligns with the moderate behavioral and biochemical outcomes observed.

As evidenced by the literature, our results demonstrated that CFA induces persistent nociceptive behaviors such as mechanical hyperalgesia, cold allodynia, and edema [51–53]. Nociceptive behaviors followed a prolonged inflammatory process triggered by inflammatory cytokines release, including TNF, IL-1β and IFN-γ, neutrophil recruitment, and oxidative damage [20, 30, 52, 54]. CFA mediates protein damage and reduces antioxidant defenses, as evidenced by increasing protein carbonyls and decreasing sulfhydryl group (groups with reducing capacity that play a crucial role in supporting antioxidant defense systems) in the PFC. CFA also reduces antioxidant defense and up-regulates intracellular inflammatory pathways peripherally, as demonstrated by decreasing the sulfhydryl group, and Akt [19] and NLRP3 inflammasome activation [20] in the paw. In addition, CFA administration induces persistent inflammatory pain, which secondarily impacts distinct components of the pain experience, including behavioral outputs related to emotional and cognitive processing, such as mechanical conflict avoidance and anxiety-like behaviors, without altering locomotor activity. Notably, Coffee Oil improved mechanical and cold hypersensitivity without altering CFA-induced edema, indicating that the formulation modulates nociception independently of vascular swelling, further reinforcing its selective, instead of global, anti-inflammatory profile.

The positive-control validation experiment using topical dexamethasone (Online Resource 1) provides important context for interpreting the magnitude and temporal profile of the effects observed with Coffee Oil. As expected, dexamethasone produced a rapid and robust reduction in CFA-induced mechanical hypersensitivity, demonstrating superior efficacy during the early stages of inflammation. In contrast, Coffee Oil generally produced more moderate effects that emerged progressively over repeated applications. Notably, in the Mechanical Conflict-Avoidance System, reductions in escape latency became more evident at later time points, suggesting a slower development of behavioral effects compared with a conventional anti-inflammatory drug. Together, these findings indicate that Coffee Oil exerts biologically measurable antihyperalgesic effects, albeit with a lower magnitude and slower onset than dexamethasone.

The absence of an effect on CFA-induced edema may further reflect limitations associated with topical delivery. Although Pentravan® is a recognized transdermal vehicle designed to enhance dermal permeation, the skin penetration, tissue distribution, and release kinetics of Coffee Oil constituents have not been characterized. Consequently, limited penetration through the skin barrier, heterogeneous diffusion of bioactive compounds, or suboptimal local bioavailability may have reduced the concentration of active constituents reaching the inflamed tissue. Importantly, the positive-control validation experiment confirmed that the CFA model is responsive to a standard topical anti-inflammatory intervention. Therefore, the comparatively modest efficacy of Coffee Oil is unlikely to result from inadequate model sensitivity and may instead reflect limitations related to topical delivery, local bioavailability, or the intrinsic activity of its constituent compounds. These factors may help explain why Coffee Oil consistently modulated nociceptive and inflammatory outcomes while exerting only modest effects on certain inflammatory endpoints, including paw edema.

Previous studies have reported the presence of depressive- and anhedonic-like behaviors after CFA administrations [55, 56]. However, in our research, we did not observe impairment in the splash and tail suspension test. This lack of impairment can be attributed to the time of evaluation, and the strain and sex of the mice experimented. Most of the previous experiments conducted have reported emotional impairments from 2 to 4 weeks after CFA injection, mainly in male Swiss and C57BL/6 mice [51, 57, 58].

Our findings demonstrated that treatment with Coffee Oil at 10%, 20%, and 30% presented an accentuated antihyperalgesic effect at mechanical stimulus. The antihyperalgesic effects of treatment with Coffee Oil 20% were more pronounced, lasting from the second application up to 11 days after CFA administration. Its effects were prolonged for up to 3 h after topic application. Treatment with Coffee Oil 20% affects different components of pain, including efficacy in reflexive measures of mechanical and thermal hypersensitivity and improvement in the mechanical conflict-avoidance test—a reliable operant assay for measuring pain-related behaviors [35]. In addition, Oil 20% had an anxiolytic effect and enhanced hedonic-like behavior after CFA-induced inflammation. These findings suggest that Coffee Oil may influence multiple dimensions of the pain experience, as reflected by improvements in selected sensory and behavioral outcomes. However, the magnitude of these effects was modest, suggesting that they may result from partial modulation of peripheral inflammatory processes rather than primary central anxiolytic or antidepressant actions.

Coffee Oil 20% exhibits its benefits through peripheral and central inflammatory-associated mechanisms. Peripherally, Coffee Oil 20% exhibited its effects by downregulating pro-inflammatory cytokines IL-1β and TNF, suppressing Akt1/2/3 phosphorylation, NLRP3 inflammasome activation, and reducing neutrophil recruitment. These results corroborate *in vitro* studies reported in the literature, suggesting that the compounds found in coffee grounds, including phenolics and chlorogenic acids, exhibit anti-inflammatory properties. These constituents have demonstrated the ability to inhibit inflammatory mediators such as IL-8, TNF, and IL-6, contributing to the modulation of the inflammatory response [25, 59]. Pro-inflammatory cytokines such as IL-1β, IL-6, and TNF, produced predominantly by activated macrophages, contribute to the process of pathological pain [60]. Both TNF and IL-1β can directly sensitize nociceptive fibers, activating pathways that increase pro-inflammatory cytokines and prostaglandin synthesis. TNF modulates microglial and astrocytic activation, blood–brain barrier permeability, glutamatergic transmission, and synaptic plasticity, while IL-1β enhances neuronal sensitivity to pain through IL-1 receptor activation, a crucial step to initiating the process of nociception [60, 61]. Importantly, the modulation observed in our study was selective: Coffee Oil reduced IL-1β and TNF and suppressed p-Akt and NLRP3 but did not significantly alter other cytokines. IL-6, IL-10, and CRP were unchanged by Coffee Oil, although these markers were not altered by CFA at this time point in peripheral tissue. Moreover, Coffee Oil did not reverse the CFA-induced increase in IFN-γ or the reduction in IL-17B. Together, these findings indicate that Coffee Oil acts on specific intracellular inflammatory nodes rather than producing a broad cytokine-level suppression, which likely underlies the partial behavioral efficacy observed.

In addition to directly inhibiting the release of inflammatory mediators and enhancing antioxidant defense, critical factors in inflammatory pain, Coffee Oil also downregulates two main intracellular inflammatory pathways upregulated by CFA. Previous studies have demonstrated that Akt activation in primary sensory neurons [19] and inflamed peripheral tissue [62] contributing to the maintenance of chronic inflammatory pain. Additionally, Coffee Oil 20% suppressed the activation of the NLRP3 inflammasome, an important protein complex responsible for the maturation of IL-1β in inflammatory arthritis [63]. This suppression led to a subsequent reduction in IL-1β levels within the inflamed paw tissue.

Centrally, treatment with Coffee Oil 20% also demonstrated antioxidant and anti-inflammatory properties in the PFC and hippocampus, two key areas associated with central pain processing [14]. In the PFC, Coffee Oil 20% restored protein carbonyl levels, preserving protein structure from oxidative stress damage that could impair enzymatic activity or increase susceptibility to proteolysis [64]. In the hippocampus, treatment with Coffee Oil 20% modulates inflammatory cytokines, increasing pro- and anti-inflammatory mediators, and strengthens antioxidant defense [47] in response to CFA-induced inflammation. While Coffee Oil 20% increases the pro-inflammatory cytokines IL-1β, IL-6, and TNF in the hippocampus, it simultaneously elevates IL-10 levels, a cytokine with anti-inflammatory properties [60]. IL-10 is a crucial anti-inflammatory cytokine that regulates immune responses and inflammation. It reduces the production of pro-inflammatory cytokines (such as TNF, IL-1, and IL-6), promotes B cell proliferation and antibody production, suppresses cellular immunity, and promotes an anti-inflammatory phenotype in macrophages [1, 60]. These mechanisms help limit the amplification of inflammation and protect tissues from damage. Notably, these central changes occurred despite CFA not altering cytokine levels in the CNS, suggesting that the hippocampal modulation observed may reflect indirect peripheral-to-central signaling rather than direct penetration of Coffee Oil constituents into the CNS.

Notably, hippocampal cytokine modulation was observed exclusively in animals receiving Coffee Oil under CFA-induced peripheral inflammation, and not in CFA-treated animals receiving vehicle or in saline controls. This pattern indicates a state-dependent and context-specific central response, rather than a tonic inflammatory or anti-inflammatory action of Coffee Oil. Peripheral inflammation can sensitize central immune networks, particularly in stress- and immune-responsive regions such as the hippocampus, increasing their susceptibility to secondary modulatory inputs [65, 66].

The concurrent increase of pro-inflammatory (IL-1β, TNF, IL-6) and anti-inflammatory (IL-10) cytokines is consistent with coordinated immunoregulatory programs described during inflammatory adaptation and resolution, in which IL-10 acts as a key counter-regulatory mediator [67–69]. Importantly, central responses to CFA are highly dependent on brain region, molecular target, and time point, and at intermediate or later stages, including Day 7, many central markers may remain unchanged despite persistent peripheral inflammation [56, 70]. Within this framework, the selective hippocampal cytokine response observed here likely reflects indirect central immunomodulation unmasked under inflammatory conditions, rather than a generalized or pathological neuroinflammatory effect.

Together, the central and peripheral findings suggest that Coffee Oil modulates interconnected redox–inflammatory pathways rather than exerting uniform antioxidant or anti-inflammatory effects across tissues. The tissue-dependent redox responses, combined with the selective modulation of cytokines and intracellular mediators, indicate that Coffee Oil influences oxidative stress primarily in regions where inflammatory activity is prominent. This selective engagement of redox and inflammatory mechanisms is consistent with the modest but reproducible improvements in nociceptive and anxiety-like behaviors, supporting the idea that Coffee Oil acts through partially convergent peripheral–central pathways that collectively shape the behavioral phenotype.

This study has some limitations, including: (a) the use of only female mice, which limits comparability with studies using males or mixed populations; (b) the lack of control for the estrous cycle, which may introduce hormonal variability; and (c) the short follow-up period (up to 7 days), which limits the observation of longer-term effects of CFA on depressive- and hedonic-like behaviors. In addition, (d) the permeation profile, tissue distribution, and release kinetics of Coffee Oil constituents from the transdermal formulation were not evaluated. Therefore, limited skin penetration, heterogeneous diffusion of the different bioactive constituents, or suboptimal local bioavailability may have contributed to the modest magnitude of the observed effects. Although the positive-control validation experiment (Online Resource 1) confirmed that the CFA model is responsive to a standard topical anti-inflammatory intervention, the extent to which formulation- and delivery-related factors influenced the efficacy of Coffee Oil remains unknown.

Although these findings in a CFA-induced inflammation model in mice provide mechanistic insights relevant to human inflammatory pain, translation to clinical settings should be approached with caution due to species differences and controlled experimental conditions. Future studies should investigate the skin permeation, tissue distribution, and pharmacokinetic behavior of Coffee Oil constituents, as well as optimized transdermal formulations and combination strategies, to determine whether improvements in local delivery can enhance therapeutic efficacy. Together, these limitations help contextualize the scope of the present findings and highlight important considerations for future preclinical and translational studies.

## Conclusions

This study provides proof-of-concept evidence that topical Coffee Oil 20% produces modest yet consistent improvements in behavioral and nociceptive outcomes associated with CFA-induced inflammatory pain. These effects appear to result from selective modulation of inflammatory and oxidative pathways rather than from broad anti-inflammatory or antioxidant actions. Coffee Oil 20% partially alleviated inflammatory pain by reducing IL-1β and TNF, limiting neutrophil recruitment, and downregulating p-Akt1/2/3 and NLRP3 activation, while exerting tissue-dependent effects on redox balance.

Although the magnitude of these effects was generally modest, Coffee Oil demonstrated a reproducible capacity to influence interconnected peripheral–central inflammatory and redox mechanisms, which collectively contributed to improvements in mechanical hypersensitivity, cold nociception, and anxiety-like behavior. Importantly, comparison with the positive-control validation experiment using topical dexamethasone suggests that Coffee Oil exerts biologically measurable effects, albeit with a lower magnitude and slower temporal profile than a standard anti-inflammatory intervention.

Taken together, these findings support the view that Coffee Oil obtained from spent coffee grounds may be better considered as a supportive or adjunctive strategy rather than a replacement for established anti-inflammatory therapies, particularly in conditions characterized by low-grade or chronic inflammation. Future studies comparing Coffee Oil with standard topical anti-inflammatory agents, optimizing transdermal delivery, and exploring combination approaches will be important to define its therapeutic relevance and translational potential.

## Supplementary Information

Below is the link to the electronic supplementary material.

Supplementary file1 (PDF 217 KB)

## Data Availability

The datasets generated and/or analyzed during the current study are not publicly available but are available from the corresponding author upon reasonable request.

## Abbreviations

ANOVA: Analysis of variance
ARRIVE: Animals in Research: Reporting In Vivo Experiments
CAT: Catalase
CFA: Complete Freund’s Adjuvant
CNPq: National Council for Scientific and Technological Development
CRP: C-Reactive Protein
TNB: 5,5′-Dithiobis-(2-nitrobenzoic acid)
CNS: Central nervous system
EDTA: Ethylenediaminetetraacetic acid
ELISA: Enzyme-linked immunosorbent assay
IL-1β: Interleukin-1 beta
IL-6: Interleukin-6
IL-8: Interleukin-8
IL-10: Interleukin-10
IL-15: Interleukin-15
IL-17: Interleukin-17
IFN-γ: Interferon-gamma
i.pl.: Intraperitoneally
LED: Light-emitting diode
MPO: Myeloperoxidase
NF-κB: Nuclear factor-kappa B
NTP: Non-thermal plasma
PBS: Phosphate-buffered saline
PFC: Prefrontal cortex
PMSF: Phenylmethylsulfonyl fluoride
ROS: Reactive oxygen species
TBARS: Thiobarbituric acid reactive substances
TNF: Tumor necrosis factor
UNESC: University of the Extreme South of Santa Catarina
UNISUL: University of Southern Santa Catarina
UV: Ultraviolet
VF: von Frey
