# Supplementary material for: Therapeutic Effects of Spent Ground Coffee Oil on Nociception, Anxiety- and Depression-Like Behaviors, and Inflammatory Mechanisms in a CFA-Induced Intraplantar Inflammation Model in Mice
Source: Mol Neurobiol. 2026 Jul 23;63(1):784. doi: 10.1007/s12035-026-06069-3 (PMC13391712; doi:10.1007/s12035-026-06069-3)
Supplement: Supplementary file 1 — Supplementary file1 (PDF 217 KB) [file 12035_2026_6069_MOESM1_ESM.pdf]

SUPPLEMENTARY INFORMATION

Journal: Molecular Neurobiology

Article Title: Therapeutic Effects of Spent Ground Coffee Oil on Nociception, Anxiety- and Depression-like Behaviors, and Inflammatory Mechanisms in a CFA-Induced Intraplantar Inflammation Model in Mice

Authors: Elisa Mitkus Flores Lins, Anelise Leal Vieira Cubas, Bruna Hoffmann de Oliveira, Eduarda Behenck Medeiros, Khiany Mathias, Scheila Iria Kraus, Daniel Fernandes Martins, Josiel Mileno Mack, Anna Paula Piovezan, Fabricia Petronilho, Josiane Budni and Franciane Bobinski*

***Corresponding Author:**
Franciane Bobinski
Experimental Neuroscience Laboratory (LaNEx)
University of South Santa Catarina (UNISUL)
Palhoça, SC, Brazil
E-mail: francianebobinski@hotmail.com

Online Resource 1. Positive-control validation using topical dexamethasone in the CFA-induced inflammatory pain model

Supplementary Methods

To validate the responsiveness of the CFA-induced inflammatory pain model to a standard anti-inflammatory intervention, an independent validation experiment was conducted using topical dexamethasone disodium phosphate (1 mg/g) as a positive control. To contextualize the magnitude of the effects observed in the main study, a group receiving Coffee Oil 20% was included under identical experimental conditions. Male Swiss mice were subjected to CFA-induced intraplantar inflammation and allocated to the following groups: Saline/Vehicle, CFA/Vehicle, CFA/Dexamethasone, and CFA/Coffee Oil 20% (n = 8 animals/group).

Mechanical hyperalgesia was assessed using the von Frey test, and mechanical avoidance behavior was evaluated using the Mechanical Conflict-Avoidance System (MCAS). The experimental timeline, treatment regimen, behavioral procedures, and statistical analyses followed the same methodological framework described in the main study. This validation experiment was designed to provide a pharmacological benchmark for contextualizing the magnitude and temporal profile of the effects observed with Coffee Oil 20%.

Supplementary Results

Positive-Control Validation of Mechanical Hyperalgesia Using the von Frey Test

To provide a pharmacological benchmark for interpreting the antihyperalgesic effects of Coffee Oil 20%, mechanical hyperalgesia was evaluated in an independent validation experiment including topical dexamethasone disodium phosphate (1 mg/g) as a positive control (Figure 1). For mechanical hyperalgesia, two-way ANOVA revealed significant effects of treatment ($p < 0.0001$), time ($p < 0.0001$), and treatment × time interaction ($p < 0.0001$), indicating that antihyperalgesic responses differed among treatments throughout the experimental period.

Before treatment initiation (24 h after CFA administration), all CFA-treated groups exhibited comparable levels of mechanical hyperalgesia, confirming similar baseline nociceptive responses. One hour after the first treatment, dexamethasone reduced CFA-induced mechanical hypersensitivity to levels comparable to those observed in non-inflamed animals ($p > 0.9999$ versus Saline/Vehicle) and significantly lower than those of the CFA/Vehicle group (mean difference = 35.0; 95% CI = 16.0 to 54.0; $p < 0.0001$). In contrast, Coffee Oil 20% produced a partial reduction in mechanical hyperalgesia at this time point and did not differ significantly from CFA/Vehicle (mean difference = 17.5; 95% CI = −1.5 to 36.5; $p = 0.0820$).

At 48 h after CFA administration, both dexamethasone and Coffee Oil significantly reduced mechanical hypersensitivity compared with CFA/Vehicle. Dexamethasone produced a marked antihyperalgesic effect (mean difference = 42.5; 95% CI = 23.5 to 61.5; $p < 0.0001$), whereas Coffee Oil induced a more modest but significant reduction (mean difference = 22.5; 95% CI = 3.5 to 41.5; $p = 0.0129$). At this time point, dexamethasone was significantly more effective than Coffee Oil (mean difference = 20.0; 95% CI = 1.0 to 39.0; $p = 0.0344$).

At 72 h after CFA administration, both dexamethasone and Coffee Oil continued to attenuate CFA-induced mechanical hypersensitivity relative to CFA/Vehicle (mean differences = 32.5 and 22.5, respectively; $p < 0.0001$ and $p = 0.0129$). However, no significant difference was detected between the two treatments ($p = 0.5209$).

Similarly, at 96 h after CFA administration, dexamethasone (mean difference = 42.5; 95% CI = 23.5 to 61.5; $p < 0.0001$) and Coffee Oil (mean difference = 23.8; 95% CI = 4.8 to 42.7; $p = 0.0075$) both significantly reduced mechanical hypersensitivity compared with CFA/Vehicle, while the difference between treatments did not reach statistical significance ($p = 0.0539$).

Together, these findings confirm that the CFA model is responsive to a standard anti-inflammatory intervention and provide a pharmacological benchmark for contextualizing both the magnitude and temporal profile of the antihyperalgesic effects observed with Coffee Oil 20%. Although the effects of Coffee Oil were generally smaller than those produced by dexamethasone, significant reductions in CFA-induced mechanical hypersensitivity were observed at multiple time points, supporting the biological relevance of the antihyperalgesic activity reported in the main study.

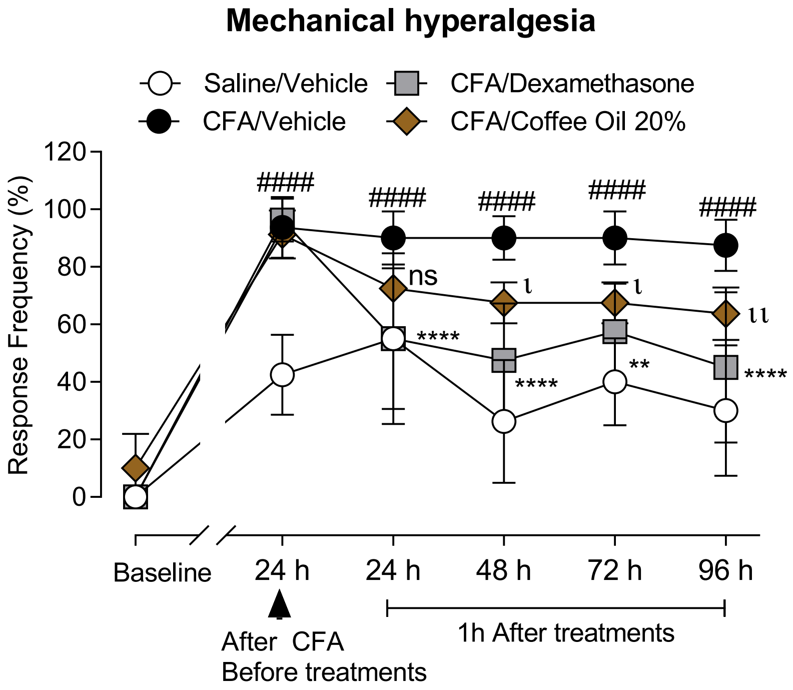

Figure 1. Positive-control validation of topical dexamethasone in CFA-induced mechanical hyperalgesia. Effects of topical dexamethasone disodium phosphate (1 mg/g) on CFA-induced mechanical hyperalgesia assessed using the von Frey test. Mechanical hypersensitivity was evaluated at baseline, 24 h after CFA administration, and 1 h after each daily treatment. Data are presented as mean ± SD (n = 8 animals/group). Statistical analysis was performed using two-way ANOVA followed by Tukey's multiple-comparison test. ####p < 0.0001 versus Saline/Vehicle; **p < 0.01 and ****p < 0.0001 versus CFA/Vehicle; ιp < 0.05, ιιp < 0.01, and ns (non-significant) versus CFA/Vehicle, as indicated in the figure.

Positive-Control Validation in the Mechanical Conflict-Avoidance System (MCAS)

As part of the positive-control validation experiment and to provide a pharmacological benchmark for interpreting the behavioral effects observed with Coffee Oil 20%, mechanical avoidance behavior was evaluated in the MCAS following treatment with topical dexamethasone disodium phosphate (1 mg/g) or Coffee Oil 20%. Assessments were performed using 2-mm and 5-mm probes on Days 1 and 4 after CFA administration (Figure 2).

For the 2-mm probe evaluated on Day 1 after CFA administration (Figure 2a), one-way ANOVA revealed a significant effect of treatment (F = 7.495, p = 0.0003). Post hoc Tukey's test demonstrated that CFA administration significantly increased escape latency compared with the Saline/Vehicle group (mean difference = 15.41 s; 95% CI = 0.58 to 30.24; p = 0.0388), confirming the development of mechanical hypersensitivity. Topical dexamethasone significantly reduced escape latency compared with CFA/Vehicle (mean difference = 27.59 s; 95% CI = 9.06 to 46.13; p = 0.0013), restoring responses to levels comparable to those observed in non-inflamed animals (p = 0.3129 versus Saline/Vehicle). In contrast, Coffee Oil 20% did not significantly alter escape latency

compared with CFA/Vehicle (mean difference = 0.71 s; 95% CI = −14.12 to 15.54; p = 0.9993). Furthermore, dexamethasone produced significantly lower escape latencies than Coffee Oil 20% (mean difference = 26.89 s; 95% CI = 8.35 to 45.42; p = 0.0018).

For the 5-mm probe evaluated on Day 1 after CFA administration (Figure 2b), one-way ANOVA revealed a significant effect of treatment ($F = 14.97$, $p < 0.0001$). CFA administration significantly increased escape latency compared with the Saline/Vehicle group (mean difference = 34.88 s; 95% CI = 9.27 to 60.50; p = 0.0036). Topical dexamethasone markedly reduced escape latency compared with CFA/Vehicle (mean difference = 79.38 s; 95% CI = 47.36 to 111.40; $p < 0.0001$), demonstrating a robust effect on CFA-induced mechanical avoidance behavior. In contrast, Coffee Oil 20% did not significantly alter escape latency relative to CFA/Vehicle (mean difference = 22.85 s; 95% CI = −2.76 to 48.47; p = 0.0964). Moreover, dexamethasone produced significantly lower escape latencies than Coffee Oil 20% (mean difference = 56.53 s; 95% CI = 24.50 to 88.55; p = 0.0001).

For the 2-mm probe evaluated on Day 4 after CFA administration (Figure 2c), one-way ANOVA revealed a significant effect of treatment ($F = 5.119$, $p = 0.0034$). CFA administration significantly increased escape latency compared with the Saline/Vehicle group (mean difference = 37.82 s; 95% CI = 10.16 to 65.49; p = 0.0035). Neither dexamethasone nor Coffee Oil 20% differed significantly from the non-inflamed control group (p = 0.9927 and p = 0.8114, respectively). Compared with CFA/Vehicle, Coffee Oil 20% significantly reduced escape latency (mean difference = 28.56 s; 95% CI = 0.90 to 56.22; p = 0.0405), whereas dexamethasone produced a reduction of similar magnitude that did not reach statistical significance after correction for multiple comparisons (mean difference = 34.25 s; 95% CI = −0.33 to 68.83; p = 0.0531). No significant difference was detected between dexamethasone and Coffee Oil 20% (p = 0.9720).

For the 5-mm probe evaluated on Day 4 after CFA administration (Figure 2d), one-way ANOVA revealed a significant effect of treatment ($F = 4.248$, $p = 0.0090$). CFA administration significantly increased escape latency compared with the Saline/Vehicle group (mean difference = 42.53 s; 95% CI = 9.26 to 75.79; p = 0.0070). Neither dexamethasone nor Coffee Oil 20% differed significantly from the non-inflamed control group (p = 0.7298 and p = 0.8889, respectively). Compared with CFA/Vehicle, Coffee Oil 20% significantly reduced escape latency (mean difference = 33.49 s; 95% CI = 0.23 to 66.76; p = 0.0478), whereas dexamethasone produced a reduction of similar magnitude that did not reach statistical significance after correction for multiple comparisons (mean difference = 26.29 s; 95% CI = −15.29 to 67.87; p = 0.3466). No significant difference was observed between dexamethasone and Coffee Oil 20% (p = 0.9675).

Overall, dexamethasone produced a stronger and earlier reduction in CFA-induced mechanical avoidance behavior, particularly during the Day 1 assessment. In contrast, Coffee Oil 20% exhibited a delayed behavioral effect that became evident on Day 4, reducing escape latency under both probe conditions. These findings further confirm the responsiveness of the CFA model to a standard anti-inflammatory intervention and provide a pharmacological benchmark for contextualizing the behavioral effects observed with Coffee Oil 20%. Although the behavioral effects of Coffee Oil were generally less

pronounced than those produced by dexamethasone, significant reductions in mechanical avoidance behavior were detected at the later assessment time points, supporting the biological relevance of the behavioral effects observed with Coffee Oil 20% in the main study.

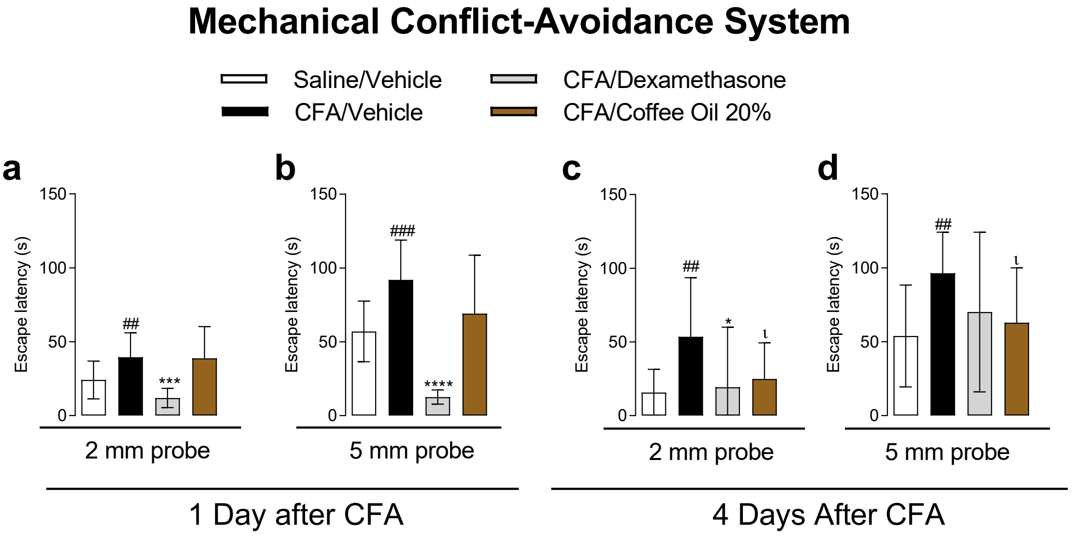

Figure 2. Effects of topical dexamethasone and Coffee Oil 20% on mechanical avoidance behavior in the Mechanical Conflict-Avoidance System (MCAS). Mechanical avoidance behavior was assessed using the MCAS with 2-mm and 5-mm probes. Escape latency was evaluated on Day 1 (a, b) and Day 4 (c, d) after CFA administration. Panels a and c show the results obtained with the 2-mm probe, whereas panels b and d show the results obtained with the 5-mm probe. Data are expressed as mean ± SD (n = 8 animals/group). Statistical analysis was performed separately for each panel using one-way ANOVA followed by Tukey's multiple-comparison test. ## $p < 0.01$ and ### $p < 0.001$ versus Saline/Vehicle; * $p < 0.05$, *** $p < 0.001$, and **** $p < 0.0001$ versus CFA/Vehicle; $^{\iota}$ $p < 0.05$ versus CFA/Vehicle, as indicated in the figure.
